# Recent Advances in Fluorescent Probes for Lipid Droplets

**DOI:** 10.3390/ma11091768

**Published:** 2018-09-18

**Authors:** Tkhe Kyong Fam, Andrey S. Klymchenko, Mayeul Collot

**Affiliations:** Nanochemistry and Bioimaging Group, Laboratoire de Bioimagerie et Pathologies, CNRS UMR 7021, Université de Strasbourg, Faculté de Pharmacie, 67401 Illkirch, France; tkhe-kyong.fam@unistra.fr (T.K.F.); andrey.klymchenko@unistra.fr (A.S.K.)

**Keywords:** lipid droplets, fluorescent probes, bioimaging

## Abstract

Lipid droplets (LDs) are organelles that serve as the storage of intracellular neutral lipids. LDs regulate many physiological processes. They recently attracted attention after extensive studies showed their involvement in metabolic disorders and diseases such as obesity, diabetes, and cancer. Therefore, it is of the highest importance to have reliable imaging tools. In this review, we focus on recent advances in the development of selective fluorescent probes for LDs. Their photophysical properties are described, and their advantages and drawbacks in fluorescence imaging are discussed. At last, we review the reported applications using these probes including two-photon excitation, in vivo and tissue imaging, as well as LDs tracking.

## 1. Introduction

**Figure 1:**
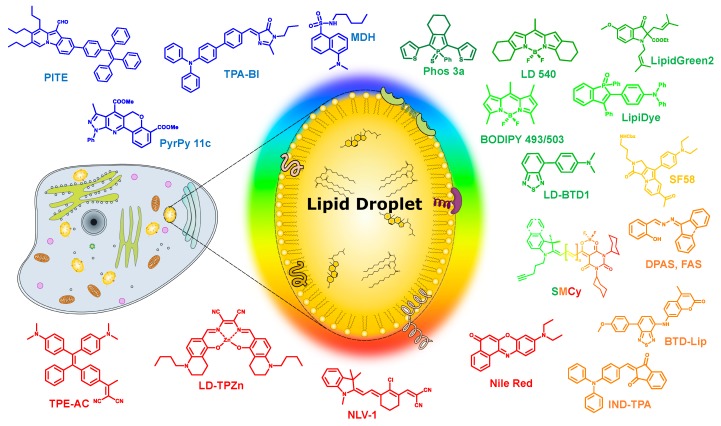
Schematic representation of a lipid droplet and the various multicolor liquid droplets (LDs) probes discussed in this review.

Lipid droplets (LDs) are the lipid storage cellular organelles consisting of a neutral lipid core, mainly containing cholesterol esters and triglycerides [1,2] covered by a phospholipid monolayer shell where a number of associated proteins were recently found (Figure 1). In particular, these include the PAT protein family, with PAT standing for Perilipin (PLIN), Adipocyte differentiation-related protein (ADRP; also called as adipophilin), and TIP47 (tail-interacting protein of 47 kDa) proteins [3]. LDs can primarily be found not only in the adipose tissue of eukaryotic organisms, but also in other types of cells, since LDs play a very important role in the regulation of cellular lipid metabolism [3].

Recently, LDs received considerable attention as they were shown to be involved in different cellular processes such as membrane formation, trafficking [4], and protein–protein interaction [5]. Moreover, the misregulation of LD functions can cause metabolic disorders such as obesity, fatty liver diseases, diabetes, atherosclerosis, and others [6]. 

In order to understand its role in different metabolic disorders and get insights on their interactions with other cellular compartments, it is of the highest importance to have reliable imaging tools for LDs. The visualization of cellular LDs can be carried out by label-free imaging techniques, such as conventional transmission light microscopy [7], direct organelle mass spectrometry [8], Raman microscopy [9], and coherent anti-Stokes Raman scattering microscopy [10]. These advanced techniques allowed us to study the biophysics of cellular LDs, but they required complex sample preparations and data analysis. In addition, the mentioned methodologies usually require cells to be fixed or LDs extraction, omitting the possibility of studying the real-time dynamics of LDs in the cellular environment. On the other hand, the fluorescence imaging techniques have proved to be powerful methods to study complex biological processes due to their high sensitivity [11]. It allows precisely imaging the localization, distribution, and biophysical properties of many intracellular compartments such as mitochondria, nucleus, plasma membrane, etc. [12,13,14].

In this review, we focus on recent advances in the field of selective LDs fluorescence imaging. In particular, the advantages and disadvantages of developed fluorescent probes to study LDs biology are discussed, as well as emerging probes that enable LD tracking in cells and tissues.

## 2. Common Methods and Probes in LDs Fluorescence Bioimaging

LD biology research is greatly assisted by chemical tools, allowing easy-to-handle manipulations to study intracellular bioprocesses in live cells. These compounds allowed scientists to image and track LDs under both physiological and pathophysiological conditions [3,15]. In this part, we will present the immunostaining targets and the commonly used molecular lipophilic dyes to identify the LDs in cells. 

### 2.1. Fluorescence Immunostaining

The immunofluorescence staining technique is a golden standard biochemical method to detect target molecules in laboratory practice. The expression of PAT proteins in LDs permits visualizing LDs using the immunofluorescence technique, in particular if adipophilin is targeted, which is usually abundant in LDs [16]. Despite being a powerful and reliable method that is applicable to staining LDs, careful considerations should be taken when immunofluorescence staining is used. Common protocols require the fixation of cells. Methods of fixation can have a dramatic impact on LDs’ morphology. In addition, some LD-associated proteins are weakly detected after the permeabilization step [16]. Thus, the detection of cellular LDs is more practical and feasible when fluorescent small molecules are used, as they stain the lipid core of native LDs. Moreover, since immunostaining requires fixation, it does not allow live cell studies and the tracking of LDs.

### 2.2. Diazo Dyes

**Figure 2:**
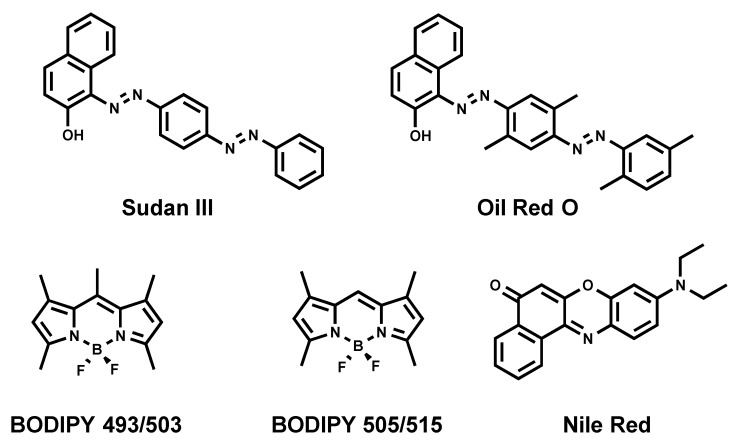
Chemical structures of commonly used fluorescent dyes.

Sudan III [17] and Oil Red O [18] (Figure 2) are diazo dyes that have been used as LD strains for decades, since they enable visualizing LDs using conventional bright-field (transmitted light) microscopy. Moreover, both dyes emit a red fluorescence, permitting the use of fluorescence microscopy to analyze LDs. The downfall of those dyes is their poor solubility, thus requiring the usage of ethanol or isopropanol for staining. Such invasive staining conditions cause the disruption of native LDs and occasional fusion, despite cells being fixed [19,20]. 

### 2.3. Nile Red

Nile Red (Figure 2) is a commercially available fluorogenic dye that is commonly used to stain LDs in cells [21]. Nile Red exhibits solvatochromic properties fluorescing in most organic solvents and a lipidic environment, while its fluorescence is diminished in aqueous media [21]. Even though Nile Red is an easy-to-handle fluorescent LD marker, there are some limitations to be taken into consideration: (i) non-specific labeling of cellular lipid organelles, in particular intracellular membranes, and (ii) Nile red has broad absorption and emission spectra that lead to cross-talk in the red channel (typically: excitation between 530–560 nm), thus making it non-suitable for multicolor imaging with different dyes or fluorescent proteins.

### 2.4. BODIPY 493/503

Another commonly used commercial fluorescent dye is BODIPY 493/503 (Figure 2) [22]. A structurally close analogue of BODIPY 493/503, namely BODIPY 505/515, is also known to stain neutral lipids [22]. Although BODIPY 493/503 rapidly and reliably labels LDs with bright green fluorescence, it possesses limitations. First, it tends to produce a background signal in imaging due to its non-fluorogenic nature. Indeed, we recently showed that BODIPY 493/503 displays a quantitative quantum yield in aqueous media [23]. Then, we also showed that BODIPY 493/503 has limited photostability [23]. At last, BODIPY 493/503 displays a small Stokes shift (the difference between excitation maximum and emission maximum), which can cause cross-talk between the excitation source and the fluorescence emission. However, BODIPYs were shown to be more selective to LDs than Nile Red, and immediately stain LDs due to a better cell permeation [24,25].

Even though Oil Red O, Nile Red, and BODIPY 493/503 proved to be useful chemical tools to visualize LDs, their utility faces the previously mentioned limitations (specificity, cross-talk, photostability, small Stokes shifts, and background noise). Moreover, the analysis should be done with high precautions; appropriate controls are necessary to be performed to discriminate false positive fluorescence signals [19]. Consequently, these limitations led to the development of new LD fluorescent probes. 

## 3. Recent Developments in LDs’ Selective Fluorescent Probes

Recent efforts have been made to develop new probes based on various lipophilic fluorophores expanding the available toolbox to probe LDs. In particular, the smart design of probes improved organelle specificity, photophysical properties, cell permeability, cell toxicity, etc. In this review, we tried to compile an exhaustive list of fluorescent probes targeting LDs spanning their fluorescence from blue to near-infrared (NIR) region. For clarity, we grouped the fluorescent probes by their reported emission color in the less polar solvent as followed: blue (λ_em_ < 500 nm), green (λ_em_ = 500–550 nm), orange (λ_em_ = 550–600 nm), and red to NIR (λ_em_ > 600 nm).

### 3.1. Blue Emitting LD Probes (λ_em_ < 500 nm)

The color availability of LD markers provides the flexibility of setting multicolor fluorescence imaging. The gap in the blue region was recently filled by the development of blue-emitting LD probes.

**Figure 3:**
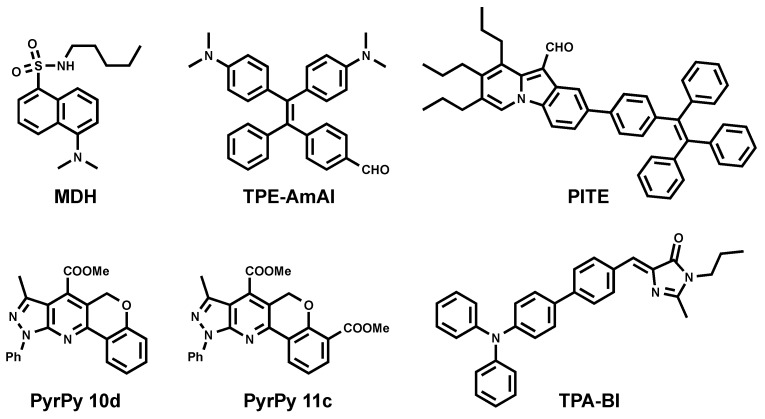
Chemical structures of blue emitting LD probes.

To this end, Yang et al. reported the utility of commercially available fluorophore monodansylpentane (MDH) as a blue marker of LDs (Figure 3) [26]. MDH was challenged to be compatible in multicolor one-photon excitation (1PE) imaging. It was also compatible with two-photon excitation (2PE) imaging using 760-nm excitation. 

Inspired by the successful utility of MDH as a blue LD marker, two series of dyes were developed based on dihydrochromenopyrrolopyridine and the –pyrazolopyridine core (Figure 3. PyrPy 10d, PyrPy 11c) as potential blue LD markers [27]. The dyes showed solvatochromic behavior. Bathochromic shifts were observed with an increasing polarity of solvents indicating a greater dipole moment in the excited state in comparison to the ground state of the molecules. This feature proved that the molecules undergo intramolecular charge transfer (ICT) and are associated with positive solvatochromism. It was noted that the introduction of the ester group into the pyridine core improved ICT and cell permeability. Moreover, the introduction of the second ester group dramatically increased selectivity toward LDs.

PITE [28] arose from the combination of pyroindole (PI) and tetraphenylethylene (TE) (Figure 3). PI emits strong green fluorescence in organic solvents, while its fluorescence is quenched in aqueous solution due to poor solubility and aggregation. On the other hand, TE is known to be an aggregation-induced emission (AIE) molecule [29]. Coupling PI and TE resulted in a molecule with new properties. Authors have reported PITE capacity to specifically detect LDs. The absorption spectra of PITE displays two bands at 320 and 420 nm as contributions of both moieties TE and PI respectively, while it emits at 490 nm and does not depend on excitation wavelength. Using *Saccharomyces cerevisiae* (as the yeast cell model) and HeLa cells (as the mammalian cell model), Sk et al. showed the compatibility of PITE as a blue fluorescent LD probe [28].

Other blue AIEgens that are specific for LDs were developed in the Tang group and named TPE-AmAl [30] and TPA-BI (Figure 3) [31]. TPE-AmAl consists of TE as an AIE-unit emitting in the blue region, the alkylamino group as an electron donor, and carbonyl as an electronacceptor to promote the intramolecular charge transfer (ICT) process. Although TPE-AmAl emits at 610 nm as aggregated form in aqueous conditions, it exhibits blue emission once in cellular LDs. This difference is attributed to the non-polar environment of LDs and solvatochoromic properties of the dye (blue shift in less polar solvents). The LD-targeting property was confirmed by costaining with Nile Red [30]. TPA-BI bears triphenylamine (TPA) moiety as an electron donor and as a common unit for enhancing two-photon absorption (2PA) properties. On the other hand, benzylidene imidazolone (BI) was shown to be AIE-active. TPA-BI exhibited solvatochromic properties emitting from blue to red when the solvent changed from n-hexane (447 nm) to acetonitrile (619 nm), which was nearly covering the full visible spectrum. Authors have shown that TPA-BI can perform both in 1PE and 2PE fluorescence cell imaging. In 1PE imaging, TPA-BI also showed an excellent resistance to photobleaching in addition to high LD specificity. Since TPA-BI shows a 2PA cross-section (δ_2PA_) value of up to 213 GM at 840 nm, the evaluation of its compatibility in 2PE cell imaging was also conducted. The result showed that TPA-BI performed better in 2PE in comparison to 1PE imaging.

Even though reported blue probes successfully stained intracellular LDs, they share common limitations such as limited brightness (due to low ε), a high background signal due to cell autofluorescence, and the considerable phototoxicity of ultraviolet/violet excitation. 

### 3.2. Green Emitting LD Probes (λ_em_ = 500–550 nm)

**Figure 4:**
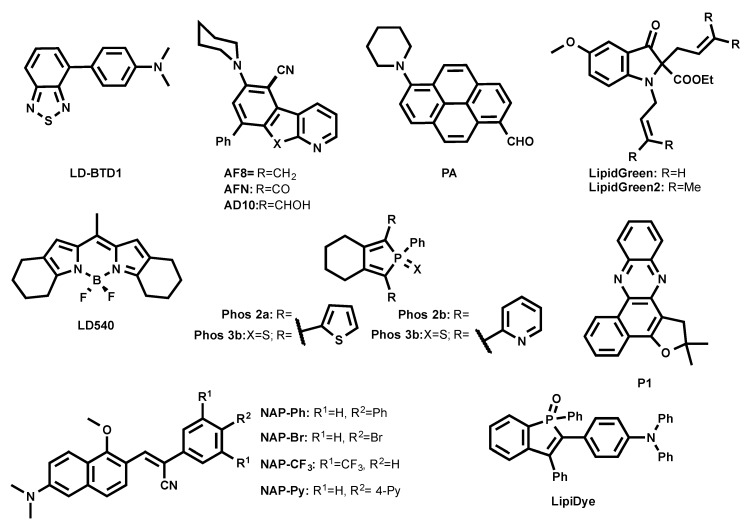
Chemical structures of green-emitting LD probes.

Anh et al. developed green-emitting LDs probes LipidGreen (λ_ex/em_ in PBS = 485/515 nm) and LipidGreen2 (λ_ex/em_ in PBS = 456/534 nm) (Figure 4) [32,33]. Both dyes were proved to stain LDs in co-localization experiments with LD-associated protein (Periplin) and red emitting LipidTOX™ (neutral lipid).

A BODIPY-based lipophilic dye LD540 was introduced for LD imaging (Figure 4) [34]. In sunflower oil, LD540 fluoresced at 545 nm. Well-defined images were obtained when LDs were costained with LD540 and LD-protein ancient ubiquitous protein 1 (AUP1). It is noteworthy that, unlike BODIPY 493/503 and due to its red-shifted emission wavelength, LD540 is prone to cause cross-talk between green and red fluorescence channels in microscopy.

Another family of fluorogenic dyes that have been reported to stain LDs are based on azafluorenes (AF8, AF10) and azafluorenones (AFN) (Figure 4) [35]. In DMSO, AF8 and AF10 showed absorption maxima at 375 nm and 352 nm, respectively, and fluorescence maxima at 479 nm and 477 nm, respectively. Interestingly, in DMSO, AFN exhibited absorption maximum at 432 nm and a fluorescence maximum at 592 nm with a Stokes shift of 160 nm. AF8 and AF10 did not exhibit solvatochromism, while AFN showed solvatochromic shifts, increasing polarity from cyclohexane (λ_em_ = 510 nm) to DMSO (λ_em_ = 592 nm). The solvatochromic nature of AFN clearly indicates that the molecule undergoes ICT process. Even though AF8 and AF10 showed interesting photophysical properties, only AFN was able to permeate cells and nicely co-localized with Nile Red, showing its specificity to LDs [35].

A family of two-photon excitable naphthalene-based LD probes abbreviated as NAP AIEgens (NAP-Ph, NAP-Br, NAP-CF_3_, and NAP-Py, as shown in Figure 4) was recently introduced [36]. AIEgens NAP probes have been reported to fluoresce at 523–540 nm in an aggregated state in aqueous solution. The NAP AIEgens exhibited a large Stokes shift (>110 nm) and good 2PA cross-section (δ_2PA_) (45−100 GM at 860 nm), and were thus compatible for 2PE fluorescence live cell and tissue imaging. The ability to specifically stain LDs at very low concentrations (50 nM) within a short time (~15 min incubation) makes NAP probes very attractive to use in the LDs’ cell and tissue imaging by the bot 1PE and 2PE fluorescence microscopy.

Recently, Appelqvist et al. introduced a new green LD selective emitter based on a benzothiadiazole (BTD) fluorophore named LD-BTD1 (Figure 4) [37]. BTD was cross-coupled to electron-donating dimethylaminophenyl, resulting in a push–pull fluorophore. In hexane, LD-BTD1 exhibits an absorption maximum at 420 nm (ε = 7100 M^−1^ cm^−1^) combined with a fluorescence maximum at 511 nm. LD-BTD1 exhibits an impressive solvatochromic shift with increasing solvent polarity from hexane (λ_em_ max = 511 nm) to DMSO (λ_em_ max = 759 nm). Since the excitation and emission spectra of LD-BTD1 overlap with Nile Red due to its broad peaks, it was challenging to proceed with co-localization experiments in a fluorescence imaging experiment. 

A polarity-sensitive benzophosphole oxide-based fluorophore was recently developed as an LD marker under commercial name LipiDye (Figure 4) [38,39]. In toluene, it absorbed at 415 nm and emitted at 528 nm, as the ICT fluorophore LipiDye exhibited solvatochromic properties. Interestingly, once LipiDye stained LDs, its emission maximum was between 521–530 nm. LipiDye also labeled other cellular compartments, but with a red-shifted emission (λ_em_ = 556–565 nm). Due to these spectral differences, it was possible to obtain deconvoluted images to discriminate the hydrophobic environment of LDs from other cellular domains. 

A similar approach was employed by Niko et al. using a push–pull pyrene probe **PA** (Figure 4) [40]. This dye features absorption around 430 nm, with a good extinction coefficient in apolar media (ε = 25,000 M^−1^ cm^−1^), a quantum yield close to unity, and emission ranging between 480–600 nm as a function of solvent polarity. Its cell permeability enabled the efficient labeling of different lipid structures inside the cells, showing the brightest signal from LDs. Due to the strong solvatochromism of PA, lipid droplets were clearly distinguished by their characteristic emission color, using fluorescence ratiometric imaging that was obtained as a ratio of red channel > 550 nm to green channel < 550 nm. In this case, LDs appeared as the most apolar intracellular lipid structures.

A series of non-fused pyridyl and thienyl-substituted phospholes was proposed as alternative green LD stains (Phos, as shown in Figure 4) [41]. It was shown that the introduction of thienyl groups to the phosphole caused shifts to the red region both in excitation and emission spectra compared to pyridyl groups. In DMSO, Phos 2b and Phos 3b exhibited excitation maxima at 377 nm and 374 nm and emission maxima at 554 nm and 550 nm, whereas Phos 2a and Phos 3a displayed excitation maxima at longer wavelengths, 439 nm and 429 nm, and emission maxima at shorter wavelengths, 470 nm and 512 nm, respectively. It was also shown that thienyl-derived phospholes (Phos 2a and Phos 3a) outperformed the pyridyl-derivatives in fluorescence LD cell staining.

Exploiting natural products as intermediates, Moliner et al. designed the phenazine P1 (Figure 4) as an LD-specific probe [42]. P1 was reported to be environment-sensitive, and could report viscosity. In dioxane, P1 absorbed at 428 nm and fluoresced at 500 nm, while it formed weakly emissive aggregates in water. P1 co-localized with Nile Red in HeLa cells.

### 3.3. Orange Emitting LD Probes (550 nm < λ_em_ < 600 nm)

**Figure 5:**
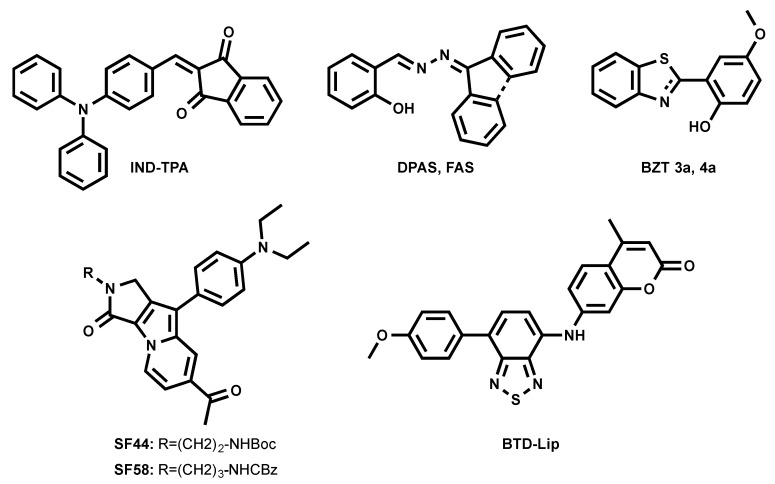
Chemical structures of orange-emitting LD probes.

A series of fluorogenic-dyes based on Seoul-Fluor (SF) core was reported to target cellular LDs (Figure 5) [43,44]. By introducing the electron-donating diethyl amino group to the SF core, the molecule was tuned to be fluorogenic, undergoing an ICT process. As consequence, a promising candidate to probe LDs was found to be SF44. The latter fluoresces in a solvatochromic manner, spanning its emission from 561 nm to 624 nm (from diethyl ether to acetonitrile). The authors confirmed that SF44 indeed stains LDs by immunostaining LD-associated membrane proteins—adipose differentiation-related protein (ADRP) and perilipin—in the differentiated 3T3-L1 cells and by co-localization experiments using Nile Red [43]. A further rational design to increase the lipophilicity of the molecule resulted in the discovery of the promising candidate SF58 [44]. The improved analogue was tested together with SF44 to probe the LDs in microalgae [44].

Tang et al. recently introduced FAS and DPAS as AIEgens reporting LDs in cells (Figure 5) [45]. They underwent an excited-state intramolecular proton transfer (ESIPT) process, giving large Stokes shift and enhanced orange and yellow emissions due to their keto forms’ fluorescence in the aggregated form. FAS and DPAS showed two emission peaks in solutions. Emission from the short-wavelength region corresponds to enol forms of the molecules, while long-wavelength emission is accredited to keto forms (λ_em_ = 430/600 nm for FAS; λ_em_ = 425/550 nm for DPAS). FAS and DPAS were nicely co-localized with BODIPY 493/503, proving their LD targeting.

The previously mentioned green-emitting BTD fluorophore was finely tuned into a BTD–coumarin hybrid resulted to discovery of new red-emitting probe, BTD-Lip (Figure 5), for LDs imaging [46]. BTD-Lip consists of BTD bearing a coumarin moiety in order to extend the π-conjugation. BTD is known to be a good electron acceptor; therefore, the p-methoxyphenyl group was linked to BTD as an electron donor to facilitate the ICT process in an excited state. ICT molecule BTD-Lip fluorescence is solvent-dependent, ranging from 585 nm to 643 nm (from hexane to DMSO). BTD-Lip can mark LDs in Caco-2 cells, together with BODIPY 493/503. Interestingly, BTD-Lip can also report LDs in worms (*Caenorhabditis elegans*) [46]. It is important to note that BTD-Lip displays broad emission bands and is prone to cross-talk in imaging.

Inspired by the interesting properties of blue LDs probe TPA-BI (Figure 3) [31], Tang et al. attempted to tune the spectral properties of the molecule by changing the electron-accepting moiety from benzylidene imidazolone to indane-1,3-dione, resulting in a new orange-emitting IND-TPA (Figure 5) [47]. In THF, IND-TPA absorbed at 478 nm and emitted at 594 nm. IND-TPA displayed atypical AIE behavior: increasing water fraction up to 70% in THF/water solution resulted in a 13.8-fold florescence intensity drop, but the further addition of water up to 90% led to a 20.4-fold fluorescence increase. The authors explained that the drop of IND-TPA fluorescence is caused by a twisted intramolecular charge transfer (TICT) effect [48], while the further fluorescence enhancement is credited to aggregation, which caused poor solubility in the water [47]. The co-localization of IND-TPA with standard LD marker BODIPY 493/503 proved the probe specificity. Unlike TPA-BI, IND-TPA gave high signal-to-background ratios. IND-TPA showed a moderate two-photon absorption cross-section of 119 GM upon excitation at 920 nm.

The first example of the photoactivatable AIE probe for LD imaging was recently reported by the same group (BZT 3a Figure 5) [49]. 2-(2-hydroxyphenyl)-benzothiazoline (BZT 3a) can be generated in situ from its disulfide precursor. Such a synthetic route simplified the availability of the LD probe and made it easy to use. BZT 3a underwent photooxidation under illumination at 365 nm to generate an oxidized form of BZT 4a. BZT 4a showed an emission peak at 570 nm in the solid state, while it was absorbed at 365 nm. Further investigations showed that in cells, BZT 3a co-localized with BODIPY 493/503. Interestingly, the photoactivation of BZT 3a could occur under two-photon illumination at 780 nm. 

Despite the limited brightness compared to commercial BODIPY 493/503, the green and orange LD markers mentioned above were shown to be chemically accessible, cell permeable, and organelle-specific. In addition, some probes have large Stokes shifts, and are two-photon excitable. On the other hand, their broad absorption and emission spectra can lead to fluorescence cross-talk in the red channel, making the utility of those dyes challenging in multicolor fluorescence imaging.

### 3.4. Red to Near-Infrared Emitting LD Probes (λ_em_ > 600 nm)

Fluorescent dyes operating in the red region are in high demand due to their compatibility with the widely used eGFP protein labeling and with biomedical diagnostics techniques. The predominant advantages of red fluorophores are: (i) low background signal from the autofluorescence of biological samples, (ii) efficient excitation of dyes in thicker tissue samples, and (iii) reduced light scattering.

**Figure 6:**
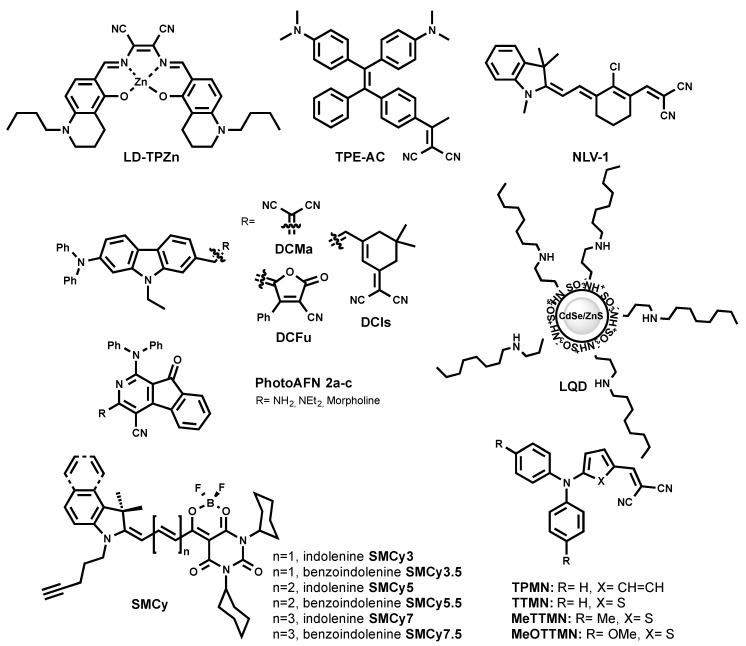
Chemical structures of red-emitting LD probes.

A red-emitting LD probe was proposed based on a luminescent Zn (II) complex with a salen ligand named LD-TPZn (Figure 6) [50]. In DMSO, LD-TPZn showed a sharp absorption band centered at 390 nm with a shoulder at 440 nm assigned to π–π* transition, and a low-energy band centered at 599 nm. It displayed an intense red emission at 637 nm and a quantum yield of 0.44. LD-TPZn displayed fluorogenic behavior, since it formed non-emissive aggregates in PBS, while there was fivefold fluorescence increase in oil-in-water emulsion. LD-TPZn was proved to stain LDs in cells by co-localization with BODIPY 493/503 and immunolabeled LD-associated protein periliplin-1 in cells. LD-TPZn was reported to undergo endocytic cellular uptake, which is an energy-dependent process [50]. The authors claimed that this property prevents non-specific interaction with other cell organelles, thus reducing background fluorescence. LD-TPZn exhibited a two-photon absorption cross-section (δ) of ca. 110 GM at 880 nm, and was used to image LDs in adipose tissue.

Recently, LQD (Figure 6) was reported as a quantum dot-based fluorescent probe that is selective for LDs [51]. LQD consisted of a red-emitting CdSe quantum dot core capped with a ZnS shell, and covered with polyacrylate coated with octyamine tails. LQD in colloidal solution showed a narrow emission peak at ~600 nm. As a quantum dot, LQD has a wide excitation window; thus, blue excitation was used for the imaging of LDs. LQD entered cell via lipid-raft endocytosis and co-localized with Nile Red. The lipid-raft endocytosis mechanism of LQD uptake minimized its trafficking to the endosome/lysosome. As the authors mentioned, the designed probe can be challenging to use in vivo, since they can accumulate in organs due to the larger size compared to the required size for the clearance.

The exploration of 2-azafluorenone core by Tang et al. resulted in the development of the photoactivatable LD-specific probes [52]. The probes are based on dihydro-2-azafluorenones that could undergo photooxidative raction to form AIEgens 2-azafluorenones (PhotoAFN 2a-c, as shown in Figure 6). They spanned their maximum fluorescence from 610 nm to 624 nm in THF solution, and from 571 nm to 620 nm in a solid state. As TICT molecules, PhotoAFN 2a-c exhibited solvatochomic properties (blue shift with the decrease of solvent polarity). As expected, in LDs, the probes emitted closer to the orange region of the wavelength. The photoactivation property allowed sequentially activating the fluorescence in the LD of selected cells. In addition, the authors showed the difference in the number of LDs in lung cancer cells and normal lung cells. 

Further efforts of Tang et al. resulted in a NIR AIEgen-based LD probe to stain LDs (TPE-AC Figure 6) [53]. By simple modification of the TE core with another electron-accepting group, malononitrile (instead of carbonyl in TPE-AmAI), the spectral properties of TE were tuned from blue emission to the NIR region, which was an AIEgen named TPE-AC. TPE-AC aggregates in water and shows a NIR emission at 780 nm, with an absolute quantum yield of 5% in the solid state. Surprisingly, unlike TPE-AmAI, there was no blue-shifted emission for TPE-AC once it stains cellular LDs, as it preserved its emission in the NIR region similar to in aqueous solution.

The redesign of the triphenylamine core led to the discovery of red/NIR AIEgens, TPMN, TTMN, MeTTMN, and MeOTTMN (Figure 6) [54]. Ranging their emission from 648 nm to 719 nm in the solid state, these AIEgens exhibited interesting characteristics such as synthetically one-pot accessible molecules, large Stokes shifts, and bright emissions. They were shown to specifically stain LDs in a co-localization experiment with lipophilic BODIPY 493/503. The reported AIEgens demonstrated the capacity to produce reactive oxygen species, and thus open the venue to be utilized as photodynamic therapy sensitizers. In addition, TPMN, TTMN, and MeTTMN enabled fluorescence imaging in live zebrafish embryos.

The same group recently reported carbazole-bridged push–pull NIR AIEgens named DCMa, DCIs, and DCFu (Figure 6) [55]. These luminogenic molecules exhibited polarity-dependent solvatochromism. In polar solvent, they are non-emissive due to the TICT effect. In aggregated form, their emission ranged from 665 nm to 775 nm. DCMa, DCIs, and DCFu stained LDs suggested by the co-localization with BODIPY 505/515. All three dyes were two-photon excitable and showed moderate photosensitizing properties to generate reactive oxygen species.

One of the latest approaches to developing a NIR LD probe resulted in the discovery of NLV-1 (Figure 6) [56]. NLV-1 is a merocyanine composed of an indolenine part and a malonitrile moiety (Figure 6). The fluorogenic property of the molecule is caused by free rotation motion in low viscous media and stabilization of the planar emissive form in highly viscous media. Owing to such a property, NLV-1 can report viscosity changes in LDs. The probe displayed a red shift in absorption from 644 nm to 680 nm (from methanol to glycerol), while weak emission at 704 nm in methanol enhanced and shifted to 719 nm in glycerol. The turn-on fluorescence effect was 13.77-fold. Even though NLV-1 can target LDs in cells, it fluoresces only when native LDs environment viscosity is increased by the treatment of viscosity modulators (Monesin, Nystatin) [56]. This property is indeed useful to study the viscosity changes of LDs, but makes it impractical to use NLV-1 as an LD label under physiological conditions.

### 3.5. Families of LD Probes with Tunable Emission Color

Recently available LipidTox™ Neutral Lipid Stains by Thermofisher were developed to characterize the intracellular accumulation of neutral lipids [57]. Unfortunately, their structural information has not yet been disclosed. LipidTox™ Neutral Lipid Stains are available in three colors: green (λ_x/em_ = 495/505), red (λ_x/em_ = 577/609), and deep red (λ_x/em_ = 637/655). The series of LDs stains was reported to be more specific to LDs in comparison to Nile Red. The drawback is the compatibility for fixed cells only.

In 2018, a family of six fluorogenic dyes named StatoMerocynaines (SMCy) was introduced (Figure 6) [23]. SMCy exhibited impressive performance as fluorescent LD-targeting probes (Figure 5) [23]. The structure of SMCy is composed of barbiturate-based merocyanine with a dioxaborine bridge. Its design included: (i) two cyclohexyl rings to increase the molecule hydrophobicity and bulkiness that can help in preventing non-specific staining; (ii) the non-charged nature favoring attraction to a neutral lipid-rich environment; (iii) the length of the polymethine chain and extension benzoindolenine moiety instead of indolenine to provide the tunability of the photophysical properties; (iv) a dioxaborine bridge to fix a degree of rotation of the fluorophore and therefore enhances its brightness, moreover; and (v) a pentynyl tail to give the opportunity to functionalize the dye via bioorthogonal chemistry.

In oil, the SMCy mostly displayed narrow absorption and emission spectra and spanned their fluorescence from yellow to the NIR (from 541 nm to 753 nm). The brightness of SMCy was solvent-dependent; they generally displayed high molar extinction coefficients up to an impressive value of 394,000 M^−1^·cm^−1^ and quantum yields of 100%. In the light of the high lipophilic nature of the SMCy dyes, their photophysical properties were measured in water and colza oil (which was mainly composed of unsaturated long chain triglycerides). Remarkably, SMCy fluorogenic dyes showed up to 1700-fold fluorescence enhancement from water to oil, which is significantly larger compared to other reported LD probes. The fluorogenic behavior could be assigned to a combination of different factors. First, the considerable ICT character of these dyes makes them more fluorescent in less polar and viscous media. Second, in water, they undergo aggregation-caused quenching, which makes them non-fluorescent in aqueous media. This unprecedented light-up effect of SMCy dyes provided a background-free fluorescence imaging of LDs. SMCy co-localized well with both Nile Red and BODIPY 493/503. Interestingly, orthogonally colored SMCy were able to cross-co-localize as well. While IND-TPA can be used for tracking the dynamic motions of LDs in live cells, whether IND-TPA is compatible in multicolor multiorganelle tracking such as SMCy was not shown.

SMCy 3 was shown to be an efficient “green” LD marker [23]. Due to its sharp excitation and emission and its high brightness (ε = 87,000 M^−1^ cm^−1^, φ= 0.25 in oil), SMCy 3 provides a robust fluorescence signal from cellular LDs in the green channel, minimizing cross-talk in the channels of other colors.

We showed that four out of six members SMCys displayed remarkably high 2PA cross-section values. Notably, SMCy 5.5 displayed 10,400 GM at 820 nm. Combined to its quantum yield close to unity in lipid-rich environments, this probe is, to our best knowledge, the brightest 2PE fluorophore [23]. Using SMCy 5.5, we were able to map LDs in mouse adipose tissue in multicolor 2PE fluorescence imaging, providing high quality images. A reconstruction of a 52-µm depth 3D 2PE image revealed clear localization of the nuclei, the LDs, as well as the collagen fibers (using second harmonic generation). Owning to their sharp absorption and emission peaks and color availability, SMCy were shown to be perfectly compatible with multicolor imaging in cells and tissues.

## 4. Application of Fluorescent LD Probes

For a long time, LDs were considered simple neutral lipids reservoirs [1,2]. Until recently, its important role as a dynamic organelle in many cellular bioprocesses, in particular metabolic bioprocesses, was reevaluated [58]. Conventional lipid dyes (Nile Red, BODIPY 493/503) permitted shining light on the elucidation of fundamental LD-associated biological processes such as the lipid biosynthesis and further storage in LDs, as well as its association with endoplasmic reticulum (ER) and communication with other cellular compartments [3,15,59]. Also, BODIPY 493/503 was able to sense nuclear LDs [60] and revealed the LDs’ biogenesis in hepatitis C-infected cells [61]. Interestingly, the sensitivity of lipophilic dyes is sufficient for monitoring the formation and degradation of LDs under stress conditions in mammalian cells [62] and bacterial pathogens [63,64]. Lipophilic BODIPY 665/676 found its utility to study the dynamics of milk LDs’ growth during mice lactation, revealing oxytocin-dependent LDs secretion [65].

Since lipid uptake by monocytes induces inflammation and this is directly connected to an elevated LD content, Nile Red served as a platform in the development of a quantitative imaging-based analysis of lipid accumulation [66]. Recently BioTek^®^ has developed a phenotypic-based assay using lipophilic dyes (Nile Red and BODIPY 493/503) to probe LDs formation in hepatic HepG2 cells associated with non-alcoholic fatty liver disease (NAFLD) caused by fatty acids overload [67]. However, Nile Red was also able to detect alterations in neutral LD content in circulating monocytes derived from patients [66] and report LDs NAFLD-associated overproduction [67].

**Figure 7:**
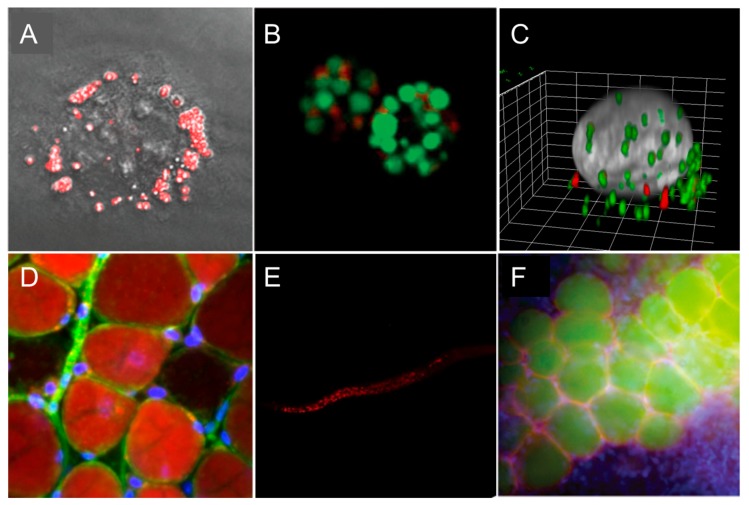
Representative fluorescence microscopy images of LDs. (**A**) Two-photon excited fluorescence image of LDs labeled with IND-TPA in HCC827 cells [47]. (**B**) LDs of green algae stained by SF44 (in green); fluorescence from chlorophyll (in red) [44]. (**C**) Three-dimensional (3D) view of a KB (HeLa derivative) cell displaying exchanged LDs from another cell (SMCy 3.5 in green and SMCy 5.5 in red) [23]. (**D**) Image of adipocytes in mouse adipose tissue (25 µm depth) obtained by a maximum projection of a Z-stack (100 slices of 250-nm depth each). LDs were stained with SMCy 3.5 (in red), nuclei in blue, and plasma membrane in green [23]. (**E**) Images of LDs stained with BTD-Lip (in red) in *C. elegans* (worm) [46]. (**F**) LDs imaging with LipidGreen (in green) in vertebral adipose tissues of zebrafish (nucleus in blue, LD-associated protein in red) [32]. Images (**A**,**B**,**F**) were reproduced with permission from The Royal Society of Chemistry. Images (**C**,**D**,**E**) were reproduced with permission from the American Chemical Society.

Emerged fluorescent chemical LDs probes enabled new discoveries in LDs research. One can mention efforts to apply them in biofuels studies (Figure 7B) [30,44]. Microalgae attracted considerable attention in biofuel research as an alternative energy source, since it is highly rich in lipid content. Lipid-abundant reservoirs in these organisms correlate with biomass productivity. SF44 [43], SF58 [44], and TPE-AmAl [30] were shown to robustly stain the algal LDs with high specificity to find high biofuel algae species. In addition, SF44 was subjected to develop a high-throughput screening (HTS) assay to find a small molecule modulator of intracellular LDs. Impressively, SF44-based screening assay led to the discovery of P8B05 that promotes LD formation in cells [43].

Some LDs probes found applications in in vivo imaging. BTD-Lip was able to selectively stain LDs in the worm, displaying a clear-cut distribution of LDs inside the organism, but not in the posterior region of the body, which is known to lack lipid-rich organelles (Figure 7E). The green-emitting LipidGreen and LipidGreen2 were successfully applied to image fat depots in zebrafish [32,33]. LipidGreen was able to monitor lipid synthesis and mobilization during the fasting and feeding cycles of zebrafish (Figure 7F). Also, LipidGreen potential was explored in drug screening for diacylglycerol acyltransferase 1 (DGAT1) inhibitors. DGAT1 is an enzyme that is responsible for the esterification of 1,2-diacylglycerol to form triglycerides, which are a main component of the LDs core; therefore, DGAT1 is a promising therapeutic target for metabolic regulations. The NIR emissive NLV-1 probe was applied to detect viscosity changes in zebrafish under the treatment of viscosity modulators and in living mice under Lipopolysaccharide-induced inflammation conditions [56]. Indeed, NLV-1 can nicely report viscosity alterations, but its non-specificity in complex living organisms may complicate the data analysis.

Two-photon excitation (2PE) microscopy became widely used as an advanced optical imaging technique for biomedical research [68]. 2PE imaging, in addition to reducing cell auto fluorescence, permits the long-term monitoring of cellular network dynamics while providing high spatio-temporal resolution in both cells and deep tissue samples. For these reasons, the inevitable demand resulted in the development of 2PE-fluorescent probes for LDs research. Hence, MDH (δ_2PA_ is not provided) [26], TPA-BI (δ_2PA_ = 213 GM at 840 nm) [31], LD-TPZn (δ_2PA_ = 110 GM at 880 nm) [50], IND-TPA (δ_2PA_ = 119 GM at 920 nm) [47] (Figure 7A), and the family of SMCy dyes (δ_2PA_= 178–13,330 GM) [23] were developed as 2PE LD fluorescent probes. Most of these dyes were able to image LDs under fatty acids-stressed conditions, the brightest to date two-photon excitable SMCy 5.5 was able to map LDs in mouse liver tissue under physiological conditions. Due to its extreme brightness and near-infrared emission, the further utilization of SMCy dyes in 2PE imaging of tissues or in vivo living animals imaging will facilitate discoveries in the field of LDs.

SMCy 3.5 was successfully used to stain large LDs in adipocytes as well as small circulating ones in the blood vessels (Figure 7D). Additionally, the color palette availability of LDs markers provides the opportunity to track the LDs of different populations within the same set of experiments (Figure 7C). Thus, SMCy dyes enabled demonstrating the occurrence of time-dependent cell-to-cell LDs exchange: it was shown that exchange took place after 48 h of intercellular interaction, which is not the case after 4 h or 18 h [23].

Table 1 sums up the photophysical properties of the reviewed LD probes as well as their applications.

## 5. Conclusions and Perspectives

As outlined above, small-molecule LD-targeting fluorescent probes have already contributed in LDs research, revealing their niche role in cellular metabolism. Significant progress has already been made in the development of new LD-specific probes, including: (1) the range of available emission colors was expanded, and now span from blue to NIR; (2) efficient LDs probes that are compatible with 2PE are now available; (3) the selectivity toward LDs was improved; (4) some photophysical properties were enhanced such as: higher brightness, photostability, and Stokes shifts. Most of described probes exhibit solvatochromic and fluorogenic properties, which can originate from different photophysical mechanisms, such as ICT, TICT, AIE, and aggregation-caused quenching [14]. First, the dyes are based on ICT processes that have a strong electron-donating group and good electron-accepting moiety (PyrPy probes [27], Seoul Fluor probes [43,44], azafluorenone [35], and probes based on benzothiadiazole core [37,46]. The other mechanism involves aggregation-caused quenching (ACQ), where the molecules, being aggregated and non-fluorescent in aqueous media, become highly emissive after binding lipid droplets in molecular form. This mechanism concerns the SMCy family and many other lipophilic LD probes. The third distinguished group utilizes two photophysical fluorescence-activating principals, twisted ICT (TICT) and AIE (the probes based on tetraphenylethylene core as an AIE-driving force [30,31,47,49,53,55]. The AIEgens are usually weakly emissive in organic solvent solutions, but they are strongly fluorescent in an aggregated state in aqueous media (as nanoparticles or solid film). While developed AIE-based bioprobes demonstrated their utility to stain LDs, they usually displayed blue-shifted emission in LDs in comparison to the emission in water, except for TPE-AC, DCMa, DCIs, and DCFu which preserved its NIR fluorescence [53,55]. The fluorescent probes described here have enabled the visualization and detection of LDs in cells, tissues, and living animals.

We anticipate that continued progress will be made to develop new LD-specific probes, such as probes that can be used in multimodal imaging techniques to increase the credibility of the obtained results. For instance, the introduction of radioactive isotopes to the core of reported LD-specific probes would open the opportunity to use a positron emission tomography (PET) imaging technique in addition to the fluorescence. In particular, the introduction of the radioisotope ^18^F would be significant, since it has longer lifetime in comparison to commonly used ^3^H and ^14^C, and is already widely used in clinics (for example, fludeoxyglucose (^18^F) is used for cancer diagnostics). Emerging advances in stimulated Raman scattering (SRS) imaging technology allowed the visualization of alkyne-tagged small molecules in live cells [69]. The alkyne tag exhibits a defined strong stretching bond signal of C≡C at ~2100 cm^−1^; therefore, SMCy dyes can potentially be used in SRS imaging for cellular LDs in addition to fluorescence. Interestingly, the nitrile C≡N bond has a characteristic peak at ~2200 cm^−1^; thus, fluorophores such as NLV-1, LD-TPZn, TPE-AC, DCMa, DCIs, TPMN, TTMN, MeTTMN, and MeOTTMN might have a potential in SRS imaging techniques. Thus, the application of the single probe for LDs in multimodal imaging settings will improve the fidelity of the acquired data.

In further efforts, it is essential to establish stronger collaborations between chemists and biologists for tuning the LD research, not only as tool-making oriented, but also as a *bona fide* solution that is oriented to better understanding the LD role in biological processes.

## Figures and Tables

**Table 1:** Photophysical properties of LDs probes and their applications.

Dye	λ_ex_/λ_em_, nm	ε, M^−1^ cm^−1^	QY	Excitation Mode	Application	Reference
**Commonly used**						
Nile Red	553/635 (MeOH)	44,000	n.r.	1P	Cells	[21]
BODIPY 493/503	493/503 (MeOH)	90,000	n.r.	1P	Cells	[22]
**Blue**						
PITE	313 ^a^/487 (MeCN)	n.r.	0.48 (MeCN)	1P	Mammalian and bacterial cells	[28]
MDH	405/570 (MeOH)	n.r.	n.r.	1P, 2P (δ_2PA_ n.r.)	Cells	[26]
TPA-BI	447/619 (MeCN)	34,000	0.22 (water 70%)	1P, 2P (δ_2PA_ = 213 GM at 840 nm)	1PE and 2PE imaging in cells	[31]
TPE-AmAl	400/470 (THF)	n.r.	0.22 (solid state)	1P	Cells and algae	[30]
PyrPy 10d	356/449 (CHCl_3_)	12,400	0.322 (CHCl_3_)	1P	Cells	[27]
PyrPy 11c	344/447 (CHCl_3_)	13,200	0.029 (CHCl_3_)	1P	Cells	[27]
**Green**						
BODIPY 505/515	505/515 (MeOH)	94,000	n.r.	1P	Cells	[22]
LipidGreen	485/515 (PBS)	n.r.	n.r.	1P	See footnote 1	[32]
LipidGreen2	456/534 (PBS)	n.r.	n.r.	1P	See footnote 2	[33]
LD540	540/545.5 (oil)	n.r.	n.r.	1P	Multicolor imaging in cells	[34]
LD-BTD1	420/511 (hexane)	7100	0.66 (hexane)	1P	Cells	[37]
AF8	380/479 (DMSO)	n.r.	0.31 (DMSO)	1P	Cells	[35]
AFN	428/592 (DMSO)	n.r.	0.17 (DMSO)	1P	Cells	[35]
AF10	356/477 (DMSO)	n.r.	0.18 (DMSO)	1P	Cells	[35]
NAP-Ph	409/523 (water)	n.r.	0.018 (water)	1P, 2P (δ_2PA_ = 100 GM at 860 nm)	1PE and 2PE imaging in cells and tissues	[36]
NAP-Br	409/525 (water)	n.r.	0.014 (water)	1P, 2P (δ_2PA_ = ~50 GM at 860 nm)	1PE and 2PE imaging in cells and tissues	[36]
NAP-CF_3_	425/560 (water)	n.r.	0.016 (water)	1P, 2P (δ_2PA_ = ~50 GM at 860 nm)	1PE and 2PE imaging in cells and tissues	[36]
NAP-Py	413/541 (water)	n.r.	0.015 (water)	1P, 2P (δ_2PA_ = 45 GM at 860 nm)	1PE and 2PE imaging in cells and tissues	[36]
LipiDye	415 (toluene)	18,700	0.94 (toluene)	1P	Cells	[38,39]
PA	434/521 (toluene)	25,000	0.95 (toluene)	1P, 2P (δ_2PA_ =35 GM at 820 nm)	Lipid order in cells	[40]
Phos 2a	439/554 (DMSO)	n.r.	n.r.	1P	Cells	[41]
Phos 3a	429/512 (DMSO)	n.r.	n.r.	1P	Cells	[41]
Phos 2b	437/554 (DMSO)	n.r.	n.r.	1P	Not applicable for cells	[41]
Phos 3b	374/550 (DMSO)	n.r.	n.r.	1P	Not applicable for cells	[41]
P1	428/500 (Dioxane)	n.r.	0.22	1P	Cells	[42]
**Orange**						
SF44	455/626 (MeCN)	n.r.	0.09 (MeCN)	1P	Cells, HTS for LD modulator	[43]
SF58	440/623 (MeCN)	n.r.	0.09 (MeCN)	1P	Cells and algae	[44]
FAS	322/600 (water) ^b^	n.r.	0.021 (solid state)	1P	Cells	[45]
DPAS	301/550 (water) ^b^	n.r.	0.03 (solid state	1P	Cells	[45]
BTD-Lip	455/624 (MeCN)	7586	0.2 (MeCN)	1P	Cells and worms	[46]
IND-TPA	478/594 (THF)	n.r.	0.069 (THF)	1P, 2P (δ_2PA_ = 119 GM at 920 nm)	1PE and 2PE imaging in cells	[47]
BZT 3a	309/576 (water)	n.r.	n.r.	1P, 2P at 780 nm	1PE and 2PE imaging in cells	[49]
BZT 4a	-/570 (solid)	n.r.	0.4 (solid state)	1P, 2P at 780 nm	1PE and 2PE imaging in cells	[49]
**Red to NIR**						
LD-TPZn	599/630 (DMSO)	10,600	0.44 (DMSO)	1P, 2P (δ_2PA_ = 110 GM at 880 nm)	1PE and 2PE imaging in cells	[50]
LQD	495/600 (colloidal)	n.r.	n.r.	1P	Cells	[51]
PhotoAFN 2a	409/624 (THF)	n.r.	0.006 (THF)	1P	Cells	[52]
PhotoAFN 2b	402/617 (THF)	n.r.	0.005 (THF)	1P	Cells	[52]
PhotoAFN 2c	400/610 (THF)	n.r.	0.008 (THF)	1P	Cells	[52]
TPE-AC	455/724 (THF)	n.r.	0.05 (solid state)	1P	Cells	[53]
TPMN	441/635 (MeCN)	n.r.	0.0021 (MeCN)	1P	Cells and zebrafish	[54]
TTMN	483/664 (MeCN)	n.r.	0.0032 (MeCN)	1P	Cells and zebrafish	[54]
MeTTMN	441/635 (MeCN)	n.r.	0.0021 (MeCN)	1P	Cells and zebrafish	[54]
MeOTTMN	499/-(MeCN)	n.r.	n.r.	1P	Cells	[54]
DCMa	478/665 (water)	n.r.	0.296 (solid state)	1P, 2P (δ_2PA_ = 394 GM at 940 nm)	1PE and 2PE imaging in cells	[54]
DCIs	510/709 (water)	n.r.	0.135 (solid state)	1P, 2P (δ_2PA_ = 548 GM at 980 nm)	1PE and 2PE imaging in cells	[55]
DCFu	538/755 (water)	n.r.	0.017 (solid state)	1P, 2P (δ_2PA_ = 887 GM at 1020 nm)	1PE and 2PE imaging in cells	[55]
NLV-1	680/719 (glycerol)	n.r.	0.204 (glycerol)	1P	See footnote 3	[56]
**Multicolor**						
*LipidTox^™^*					Analysis of steatosis	[57]
green	495/505	n.r.	n.r.	1P		[57]
red	577/609	n.r.	n.r.	1P		[57]
deep red	637/655	n.r.	n.r.	1P		[57]
*SMCy family*					See footnote 4	[23]
SMCy3	512/541 (oil)	82,900	0.21 (oil)	1P, 2P (δ_2PA_ = 178 GM at 690 nm, DMSO)		[23]
SMCy3.5	530/559 (oil)	100,000	0.4 (oil)	1P, 2P (δ_2PA_ = 2400 GM at 760 nm, DMSO)		[23]
SMCy5	618/648 (oil)	256,000	0.6 (oil)	1P, 2P (δ_2PA_ = 6250 GM at 740 nm, DMSO)		[23]
SMCy5.5	638/662 (oil)	169,000	0.74 (oil)	1P, 2P (δ_2PA_ = 13330 GM at 770 nm and 10400 GM at 820 nm, DMSO)		[23]
SMCy7	692/744 (oil)	44,000	0.42 (oil)	1P		[23]
SMCy7.5	716/753 (oil)	103,000	0.19 (oil)	1P		[23]

n.r. not reported. ^a^ For tetraphenylethylene (TE) region; ^b^ Emission of keto form. Footnote 1: Imaging in cells and zebrafish. Monitoring lipid synthesis and mobilization during fasting and feeding cycles. Drug screening for diacylglycerol acyltransferase 1 (DGAT1) inhibitor. Footnote 2: Imaging in cells and zebrafish. Early identification of hepatosteatosis. Footnote 3: Viscosity changes in cellular LDs, zebrafish, and mice. Footnote 4: Multicolor imaging of cells and tissues. LD tracking and intracellular exchange.
